# ADEPT: Autoencoder with differentially expressed genes and imputation for robust spatial transcriptomics clustering

**DOI:** 10.1016/j.isci.2023.106792

**Published:** 2023-05-03

**Authors:** Yunfei Hu, Yuying Zhao, Curtis T. Schunk, Yingxiang Ma, Tyler Derr, Xin Maizie Zhou

**Affiliations:** 1 Department of Computer Science, Vanderbilt University, Nashville, TN, USA; 2 Department of Biomedical Engineering, Vanderbilt University, Nashville, TN, USA; 3 Data Science Institute, Vanderbilt University, Nashville, TN, USA

**Keywords:** Automation in bioinformatics, Data processing in systems biology, Transcriptomics

## Abstract

Advancements in spatial transcriptomics (ST) have enabled an in-depth understanding of complex tissues by quantifying gene expression at spatially localized spots. Several notable clustering methods have been introduced to utilize both spatial and transcriptional information in the analysis of ST datasets. However, data quality across different ST sequencing techniques and types of datasets influence the performance of different methods and benchmarks. To harness spatial context and transcriptional profile in ST data, we developed a graph-based, multi-stage framework for robust clustering, called ADEPT. To control and stabilize data quality, ADEPT relies on a graph autoencoder backbone and performs an iterative clustering on imputed, differentially expressed genes-based matrices to minimize the variance of clustering results. ADEPT outperformed other popular methods on ST data generated by different platforms across analyses such as spatial domain identification, visualization, spatial trajectory inference, and data denoising.

## Highlights

• We developed a graph-based clustering tool for spatial transcriptomics data, ADEPT
• ADEPT selects differentially expressed genes to perform imputation
• ADEPT achieved robust clustering results with low variance
• ADEPT improved accuracy over a range of existing methods

## Graphical abstract

**Figure:**
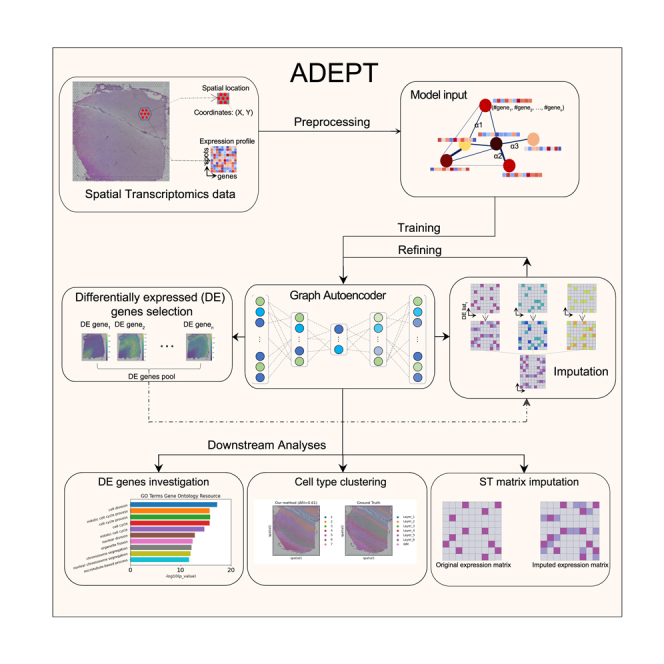

## Introduction

The complex tissues in our body consist of diverse cell types, each specialized to carry out a particular function. Cell behavior is influenced by the surrounding environment, including signaling with adjacent and distant cells.^1^ Deciphering the spatial domains of different cell types in tissue is consequently critical for understanding the behavior of cells and the progression of disease pathology.^2^ Single-cell RNA sequencing techniques (scRNA-seq) have made it possible to characterize cells by their types and physiological properties at an unprecedented per-cell resolution;^3^ however, the lack of information regarding the spatial location of cells prohibits us from investigating the complicated transcriptional architecture of heterogeneous tissues. Conducting scRNA-seq while also identifying the spatial context of cells within the tissue could allow a deeper understanding of location-specific gene expression and cell behavior. Recent technological advances in Spatial Transcriptomics (ST) have made this possible.^3^ The technique of ST has greatly accelerated the study of complex transcriptional architecture within heterogeneous tissue, while only slightly sacrificing cell resolution (1–10 cells in each sequencing spot).^4^ There are two main categories of methods of ST sequencing. The first category is performed via fluorescence-based *in situ* transcriptomics, which includes methods like single-molecule fluorescent *in situ* hybridization (smFISH),^5^ spatially resolved transcript amplicon readout mapping (STARmap),^6^ and multiplexed error-robust fluorescence *in situ* hybridization (MERFISH).^7^ The second category consists of a combination of spatial barcoding and next-generation sequencing. Methods like Slide-seq^8^ and 10x Visium are in this category^9^.

An important first step in ST research is to cluster the spots and define spatially coherent regions in terms of expression data and location adjacency. This is essential for downstream analyses, such as cell type or tissue annotation, new cell type identification, spatially variable gene identification, and gene ontology (GO) analysis.^10^ Some naive approaches which have been previously applied to ST data include traditional clustering algorithms like Louvain,^11^ spectral clustering^12^ and k-means.^13^ These methods, though capable of leveraging the spatial and histology data^14^^,^^15^^,^^16^^,^^17^^,^^18^^,^^19^ to identify segmented or layered spatial domains for different ST data, produce results which are somewhat unstable and of great variance. In recent years, several popular methods including BayesSpace,^17^ SpaGCN,^14^ SEDR,^18^ CCST,^16^ and STAGATE^19^ have been proposed and demonstrated their superiority compared to previous baseline models, however, the performance of existing clustering methods varies across evaluation criteria, experimental protocols, datasets, and downstream analyses. There is no consensus about which clustering method is the best.

Data quality across different ST sequencing techniques and types of datasets appear as a crucial factor that influences the performance of different methods and influences benchmarks. For example, previous studies with scRNA-seq data have attempted to solve the dropout effect, i.e. the large percentage of missing events or excessive zero counts,^20^ to improve the quality of the sequencing data. Gene dropout describes the phenomenon of a gene being observed at a certain expression level in one cell, but not being detected in another cell of the same type. Previous analyses of scRNA-seq data have shown that effective imputations for dropout effects could improve the clustering results and downstream analyses.^21^^,^^22^ A recent benchmarking study showed that examining and understanding the statistical properties of the excessive zero values in ST data is important to facilitate the development of best practices for various data analytic tasks in the field.^23^

In this study, we were motivated to develop a robust clustering algorithm, called ADEPT, an Autoencoder with Differentially Expressed genes and imputation, by tackling the data quality effect across different types of ST data. ADEPT employs a graph autoencoder to learn the low-dimensional latent embedding of each spot via both gene expression and spatial context. To control and stabilize data quality, ADEPT relies on the selection of differentially expressed genes (DEGs) and imputation of the multiple DEG-based matrices for the initial and final clustering of the graph autoencoder backbone, to minimize the variance of clustering results. The DEG selection and imputation are performed multiple times and averaged for estimating robustness. We have benchmarked ADEPT against five other popular methods on ST data generated by different ST platforms to demonstrate its robustness and superiority in different downstream analyses such as spatial domain identification, visualization, spatial trajectory inference, and data denoising.

## Results

### The overall pipeline of ADEPT

**Figure 1:**
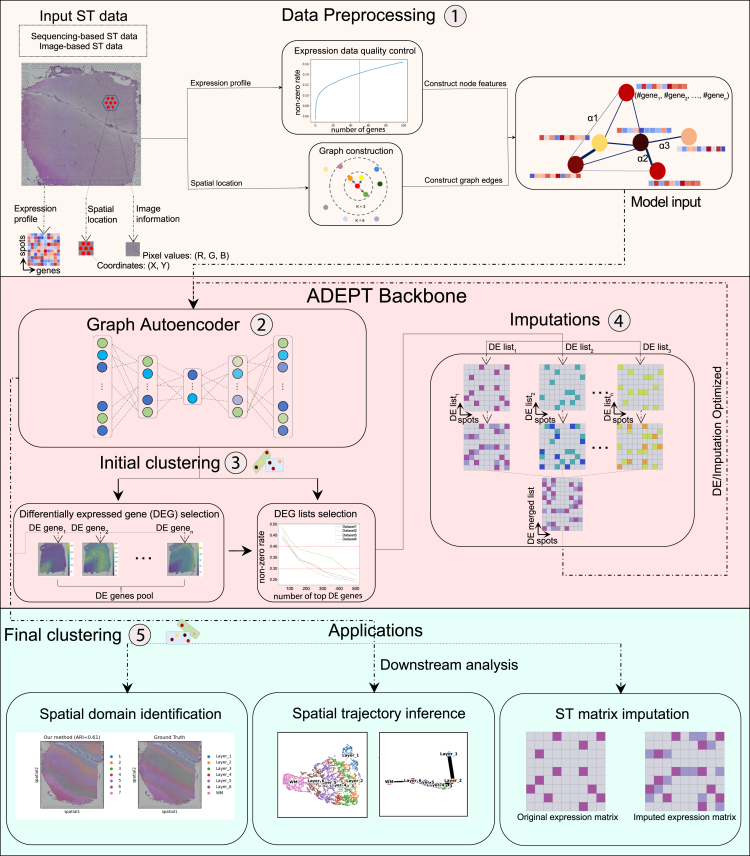
Overview of the ADEPT framework ADEPT begins by processing ST raw data as input. It first constructs a k-Nearest Neighbor graph based on spatial locations. To construct node features from gene expression information, a data quality control step is involved to remove low-quality genes. The model input is then generated and fed into the ADEPT backbone. ADEPT learns a low-dimensional latent representation with both spatial context and expression information via a graph autoencoder. After acquiring the initial clustering results from the embeddings, DEG selection, and matrix imputation modules are executed to refine the final clustering result. The output of ADEPT is then used for downstream analyses such as spatial domain identification, spatial trajectory inference, and ST matrix imputation.

ADEPT is a multi-stage framework that performs spot clustering iteratively, and gradually increases clustering quality in an unsupervised manner, without the need for label information. Its overall pipeline is described in the following steps (Figure 1): There are two types of input data for ADEPT, sequencing-based, and image-based ST data. The image-based input is optional. In the first data preprocessing step, ADEPT utilizes the gene expression profile. Each spot is treated as one node and the associated gene expression as a feature vector. ADEPT then constructs the graph structure based on the node (spot) adjacency via the k-Nearest Neighbor (kNN) algorithm. In the second step, ADEPT feeds the constructed graph to a Graph Autoencoder (GAE), to learn a low-dimensional latent representation of each node.^24^^,^^25^ The autoencoder contains a naturally coupled reconstruction loss function which is minimized to acquire the low-dimensional spatial distribution of the node embeddings, in an unsupervised fashion. In the third step, an initial clustering is performed on the node embeddings and several differentially expressed gene (DEG) lists are selected from the initial clusters relying on non-zero rates of the expression matrix. A Gaussian mixture model is used to cluster the node embeddings after the reconstruction loss threshold is reached and convergence of the model has occurred. In the fourth step, ADEPT extracts and imputes multiple DEG-based gene expression matrices, and combines them into a final imputed matrix as the GAE model input for final clustering. Finally, in the fifth step, further downstream analyses can be performed based on the final clustering result, such as spatial domain identification, spatial trajectory inference, and ST matrix imputation.

To evaluate the performance of our spatial clustering method, we used ADEPT on several annotated benchmark datasets, with five other tools. The accuracy and robustness of ADEPT were evaluated by using the adjusted rand index (ARI), Fowlkes–Mallows score (FMS), and purity, as well as comparing visualization of spatial domain identification between each tool and the ground truth. We also performed further downstream analyses such as spatial trajectory inference and ST matrix imputation for some specific datasets.

### Benchmark datasets

We used datasets from the dorsal lateral prefrontal cortex (DLPFC), a human breast cancer dataset, and the STARmap dataset.

The DLPFC dataset includes 12 human DLPFC sections, taken from three individual samples.^26^ The total number of spots ranges from 3498 to 4789, depending on the section. For this dataset, the authors have meticulously manually annotated all 12 sections for cortical layers 1 to 6 and white matter (WM).^26^ The breast cancer dataset contains 2 sections; however, only the first section of the dataset (BC1 in the article) has annotation.^18^ In the annotation provided by SEDR, the tissue is segmented into 20 areas. The breast cancer dataset contains 3,798 spots in total and around 33K genes. The STARmap dataset, which was generated from the mouse visual cortex that spans from the hippocampus to the corpus callosum, and encompasses six neocortical layers, contains just one slice. The STARmap dataset^6^ has fewer cells and sequenced genes (1020 genes on 1207 cells) when compared to the used 10x Visium datasets, but has single-cell resolution.

### Experimental setup and default parameters for ADEPT

For ADEPT, we used the Adam optimizer^27^ to minimize the reconstruction loss with an initial learning rate of $1e^{-3}$ and a weight decay of $1e^{-5}$. The default number of iterations was set to 1000. We used a 2-layer structure for both the encoder and the decoder in ADEPT’s backbone. Attention mechanisms were turned on for the first layer of the encoder and the last layer of the decoder correspondingly. The input feature dimension of the encoder’s first layer was equal to the total number of genes after filtering and data quality control. The dimension of the encoded hidden features, used for clustering spots, was set to 32. The input dimension of the hidden layer was set to 512. Because this tool uses an autoencoder backbone, the input feature size of the encoder layers was equal to the output feature size of the decoder layers, and vice versa. We run all experiments with the same default parameter setting.

### Control data quality by non-zero rate

**Figure 2:**
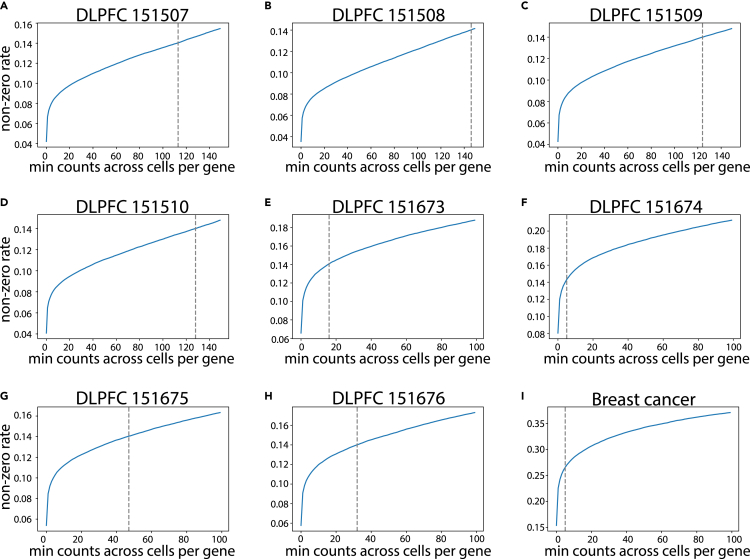
Data control plots for nine benchmark datasets (A–H) These curves depict a trend of a non-zero rate of increase as a function of an increasing number of low-quality genes being filtered in different DLPFC sections. (I) The curve depicts a trend of a non-zero rate of increase as a function of an increasing number of low-quality genes being filtered for the breast cancer dataset. The dashed line represents the hyperparameter minimum counts across cells per gene in ADEPT, which varies in different datasets.

There are two additional self-adaptive hyperparameters that need to be estimated in our framework. The first one is the minimum counts across cells per gene used in the data preprocessing step of ADEPT. In Figure 2, there are nine subplots in total for the eight DLPFC datasets and the breast cancer dataset. The figure depicts a trend of a non-zero rate of increase as a function of an increasing number of low-quality genes being filtered. In these log-like curves, there is a rapid increase near the origin, which illustrates that there are always a few genes that have significantly poor sequencing quality in most spots. The expression for these genes could contain noise or excessive zero values that would negatively affect the clustering performance, so we need to exclude these genes from our framework. Furthermore, as suggested by the starting non-zero rate in each subplot, data quality in different batches or different types of datasets could vary from each other drastically. In ADEPT, we have chosen an empirical threshold of 0.14 to ensure that after the screening of low-quality gene features, expression matrices would have acceptable and fairly uniform data quality. This approach works with high-quality sequencing data as well. In Figure 2I, the sequencing quality is high, so ADEPT would only need to remove genes with sum of counts in all spots less than 5. For image-based ST datasets generated by STARmap technology, ADEPT chooses not to execute this step by default because the number of genes is already quite lower than 10x Visium data. The minimum-counts hyperparameter is thus estimated based on each dataset and its sequencing platform.

Another crucial hyperparameter is DEG lists kept after initial clustering. As we describe in the STAR Methods section, ADEPT selects DEG lists by the non-zero rate for each dataset. DEG lists and their corresponding non-zero rates from DEG-based matrices are illustrated in Table S1. As shown in the table, each dataset has several different DEG lists that satisfy the non-zero rate threshold. All of these candidate lists will later be used in the imputation step of our framework.

### ADEPT demonstrates robust clustering performance across different ST datasets

To quantitatively demonstrate the spatial clustering capability of ADEPT, we first tested it on two 10x Visium benchmark datasets which contain manual annotations as ground truth, the DLPFC dataset and the breast cancer dataset. Using the ground truth, we compared the clustering performance of ADEPT with five other recently developed spatial clustering tools (BayesSpace,^17^ SpaGCN,^14^ SEDR,^18^ CCST^16^ and STAGATE^19^) based on the adjusted rand index (ARI), which is a commonly used similarity measure between two given clusters by considering all pairs of samples. We visualized results using SCANPY.^28^

**Figure 3:**
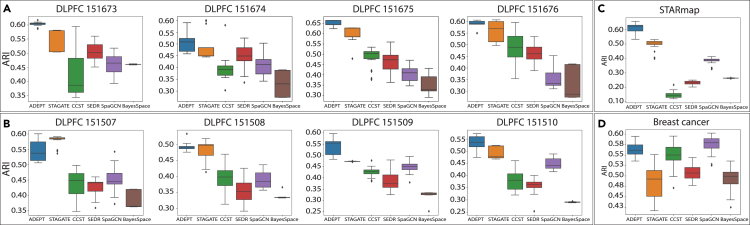
Boxplots of adjusted rand index (ARI) of six tools (A and B) ARI values for 8 DLPFC sections. (C) ARI values for the STARmap dataset. (D) ARI values for the breast cancer dataset. In the boxplot, the five horizontal lines from bottom to top denote the minimum, lower quartile, median, upper quartile, and maximum, respectively. The scattered points outside the boxplot are outliers, which refer to either extremely high or low performance.

Boxplots of ARI values from 20 experiments of each tool for all 10 datasets are shown in Figure 3. The average ARIs can be found in Table 1. For both boxplot results and average ARIs, ADEPT achieved the best performance in eight out of ten datasets, and came in second place for the other two datasets. In addition, for all four DLPFC sections in Figure 3A, from 151673 to 151676, ADEPT outperformed STAGATE, which is also an autoencoder-based method. Finally, the overall variance of ADEPT was much lower than the other methods due to the integration of the imputation step in ADEPT. In DLPFC 151507 and breast cancer, ADEPT failed to achieve the best performance; however, its result was only slightly lower than the average ARI of SpaGCN on the breast cancer dataset (Figure 3D). We also adopted two additional metrics, purity and Fowlkes–Mallows Score (FMS), to evaluate the clustering performance of all tools on these benchmark datasets (Table 2). ADEPT still accomplished the best performance in eight out of ten datasets and came in second place for the other two datasets.

**Table 1:** Table of ARI comparisons between ADEPT and five other popular methods across benchmark datasets

Methods			151507	151508	151509	151510	151673	151674	151675	151676	BC1	STARmap
ADEPT	ARI		0.543	**0.492**	**0.537**	**0.534**	**0.603**	**0.510**	**0.649**	**0.586**	0.563	**0.611**
	setting	selected DEG lists	150-250	100-150	150-200	100-150	150-400	300-450	100-250	150-250	-	25,50
		min count	113	146	124	128	16	5	47	32	5	0
STAGATE	ARI		**0.572**	0.479	0.473	0.489	0.537	0.486	0.589	0.562	0.481	0.496
CCST	ARI		0.444	0.400	0.434	0.390	0.455	0.385	0.492	0.505	0.549	0.147
SEDR	ARI		0.434	0.352	0.376	0.356	0.485	0.452	0.451	0.474	0.507	0.230
SpaGCN	ARI		0.461	0.382	0.455	0.446	0.465	0.403	0.400	0.340	**0.572**	0.379
BayesSpace	ARI		0.380	0.338	0.323	0.291	0.460	0.320	0.352	0.335	0.489	0.262

The chosen value for the hyperparameter minimum counts across cells per gene and selected DEG lists for each dataset are also provided. The highest average ARI in each column is highlighted.

**Table 2:** Table of Purity and Fawlkes Mallows Score (FMS) comparisons between ADEPT and five other popular methods across benchmark datasets

Methods	Metrics	151507	151508	151509	151510	151673	151674	151675	151676	BC1	STARmap
ADEPT	Purity	**0.836**	0.709	**0.755**	**0.755**	**0.821**	**0.755**	**0.774**	**0.726**	0.655	**0.824**
	FMS	**0.669**	0.614	**0.614**	**0.639**	**0.713**	**0.697**	**0.683**	**0.613**	**0.610**	**0.677**
STAGATE	Purity	0.691	**0.737**	0.717	0.690	0.743	0.670	0.709	0.636	0.580	0.555
	FMS	0.592	**0.646**	0.596	0.571	0.637	0.609	0.607	0.552	0.536	0.406
CCST	Purity	0.756	0.693	0.630	0.640	0.718	0.617	0.747	0.718	**0.668**	0.578
	FMS	0.558	0.527	0.558	0.529	0.548	0.505	0.617	0.578	0.585	0.318
SEDR	Purity	0.666	0.577	0.619	0.617	0.715	0.665	0.686	0.693	0.596	0.552
	FMS	0.527	0.476	0.521	0.505	0.590	0.542	0.554	0.552	0.545	0.353
SpaGCN	Purity	0.694	0.620	0.681	0.667	0.674	0.574	0.617	0.556	0.657	0.461
	FMS	0.551	0.503	0.573	0.570	0.556	0.502	0.502	0.461	0.606	0.496
BayesSpace	Purity	0.620	0.532	0.578	0.545	0.666	0.536	0.582	0.555	0.537	0.441
	FMS	0.491	0.453	0.468	0.438	0.552	0.440	0.455	0.441	0.528	0.379

The highest average purity and FMS in each column are highlighted.

**Figure 4:**
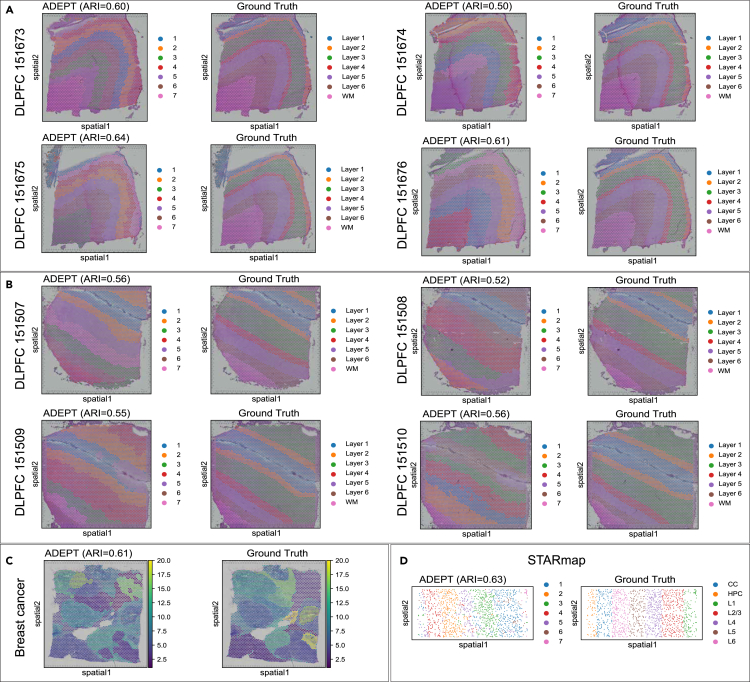
ADEPT robustly recovers the layered patterns of cerebral cortical tissue and captures the clustered distribution of tumor tissues (A and B) Visualization of 8 DLPFC sections. (C) Visualization of breast cancer dataset. (D) Visualization of STARmap dataset. Manual annotations for each dataset served as ground truth.

**Figure 5:**
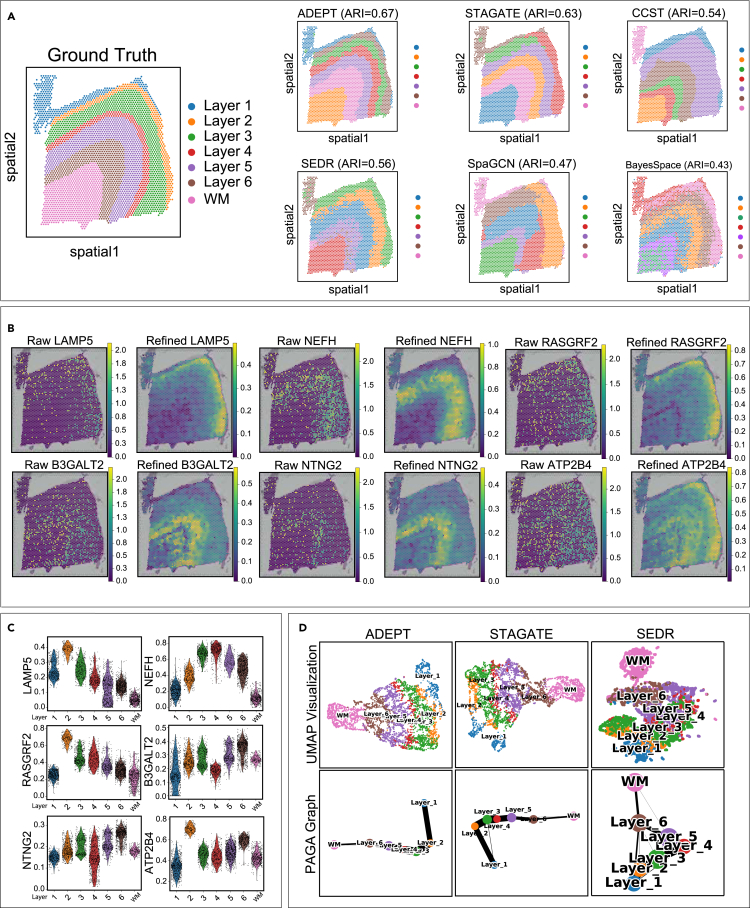
ADEPT improves spatial domain identification, enhances the spatial patterns of layer-marker genes, and correctly infers the spatial trajectory on DLPFC section 151675 (A) Best clustering results from six tools. Each visualization result has seven different clusters, corresponding to six different cortical layers and white matter, as suggested by the ground truth. (B) The imputation module of ADEPT enhances the spatial patterns of six-layer marker genes. (C) Violin plots of six-layer marker genes in b based on ground truth layer labels. (D) UMAP visualizations, and PAGA graphs for each cluster are generated respectively by ADEPT, STAGATE, and SEDR.

As expected, ADEPT can effectively distinguish spatial domains, no matter if they are in layered structures (Figures 4A, 4B, and 4D for DLPFC and STARmap datasets) or in more complex structures (Figure 4C for the breast cancer dataset). For example, in Figure 5A, in the visualization comparison of DLPFC 151675, ADEPT showed a clear pattern of separation of the seven-layered regions and achieved the best clustering accuracy (ARI = 0.64). For comparison, the clustering result of SEDR, SpaGCN, and BayesSpace could not thoroughly reveal the expected layer pattern in this section, and the border of the clusters is chaotic with many outliers inside each cluster, which impairs the overall clustering accuracy and harms the overall visual effects. CCST and STAGATE were also effective in creating clear borders between clusters; however, CCST failed to identify all seven layers consistently even with numerous runs of the algorithm.

For the mouse visual cortex STARmap dataset (Figure 4D), ADEPT achieved the best clustering performance (average ARI = 0.611) compared to the other five methods, whereas STAGATE ranked second (average ARI = 0.496).

Because some tools can achieve the best performance for certain datasets, it is hard to claim which tool works the best for every dataset. We further tested the robustness of ADEPT against five other methods by calculating the sum of rankings and the average ranking for all datasets. Each method was assigned a ranking score from 1 to 6 based on the metrics ranking. In Table S2, we present the sum of rankings and the average ranking in each dataset for all the methods. The table shows that CCST, SEDR and SpaGCN have a similar sum of rankings and average ranking. In both ranking metrics, ADEPT achieves the best average ranking (1.2), followed by STAGATE (2.3), SpaGCN (3.5), CCST (4.0), SEDER (4.2), and BayesSpace (5.5).

In terms of execution time and memory usage, ADEPT required an average runtime of 17 min for standard 10x datasets with less than 5k spots and 1.5 min for the STARmap dataset. Among all six tools (Table S3), STAGATE, SpaGCN, and BayesSpace achieved the minimum computation cost (an average runtime of 3–5 min for 10x Visium datasets, and 1.5–2 min for the STARmap dataset). All tools consumed approximately 0.5–2 GB GPU memory for 10x datasets.

### ADEPT imputes and denoises gene expressions of biomarker genes for better spatial expression patterns

ADEPT can also impute and denoise gene expressions for clear spatial expression patterns, because imputation is an innate feature of the pipeline. We compared the gene expressions of six layer-marker genes^26^ (LAMP5, NEFH, RASGRF2, B3GALT2, NTNG2, and ATP2B4) between raw data and after imputation by ADEPT in the DLPFC section 151675. In Figure 5B, the heatmaps of the raw and imputed data of the six genes are shown. Before our imputation, the heatmaps of these six genes were comparably chaotic. Using LAMP5 as an example, all spots with higher gene expression are in a scattered distribution without any significant patterns. The refined expression of LAMP5 successfully reveals the regional pattern that has a higher expression level in layer 2 and adjacent layers. We also used violin plots to confirm the same conclusion. We plotted the expression level of the same set of genes as in Figure 5B, by ground truth layers. The plot clearly shows the distinct expression levels of all six genes in different layers, which is consistent with the conclusion we have drawn previously. As reported in the previous studies,^26^^,^^29^ after imputation, these results collectively demonstrate the ability of ADEPT to reduce both noises and dropouts and recover spatial patterns more ideally, whereas its raw spatial expression, though affected by sequencing qualities and other factors, is randomly distributed.

### ADEPT improves spatial trajectory inference

In Figure 5D, we show that ADEPT was capable of revealing the distance between spatial domains by projecting embedded features down to a two-dimensional space by a UMAP plot, and further inferring the spatial trajectory using a trajectory inference tool called PAGA.^30^ For instance, in the DLPFC section 151675, those clusters of each layer were distributed reasonably and showed consistent spatial trajectories from layer 1 to layer 6 and white matter (WM) in the UMAP plots generated by the embeddings of ADEPT and STAGATE. However, in the UMAP plot of another autoencoder-based method, SEDR embeddings, layers were not separated and connected clearly, which resulted in comparably worse results. The PAGA graphs of both ADEPT and STAGATE embeddings showed a linearly-connected development tendency from layer 1 to layer 6 and WM, whereas the PAGA results of SEDR embeddings were mixed for some middle layers. CCST, SpaGCN, and BayesSpace could not be used to perform these analyses.

## Discussion and conclusion

In this study, we have proposed a multi-stage graph-based deep clustering method, combined with DEG discovery and expression profile imputation. Our method, ADEPT, was successfully tested on 10x DLPFC, Breast cancer datasets, and the STARmap dataset. It was compared with other methods in terms of visualization and metrics and biological downstream analysis, illustrating its advantages. We show that ADEPT could robustly recover the layered patterns of DLPFC and STARmap datasets, while it could also capture the clustered distribution of tumor tissues in the breast cancer dataset. By taking the advantage of DEGs and imputation, ADEPT exhibited far less prediction variance compared to other notable methods. ADEPT achieved clustering performance with the highest ARI values among five other state-of-the-art ST clustering tools for most of the datasets. In conclusion, ADEPT is a powerful and efficient method that can facilitate clustering-based downstream analyses, promote the discovery of gene markers, and refine ST expression profiles.

### Limitations of the study

As more ST clustering algorithms are implemented there is no consensus on which clustering method is the best, and a comprehensive benchmarking framework becomes increasingly necessary. Although we benchmarked several ST datasets and ADEPT outperformed existing methods most of the time, it is hard to conclude that ADEPT will achieve the best performance on other new datasets. In addition, the scalability of ADEPT is not satisfactory at present. Runtime will greatly increase for future datasets with substantially more gene features and clusters. These are areas of future improvement for our method.

## STAR★Methods

### Key resources table

**Table:** 

REAGENT or RESOURCE	SOURCE	IDENTIFIER
**Deposited data**		

Dorsal Lateral Prefrontal Cortex	Maynard et al.^26^	10x Visium
Human Breast Cancer	Fu et al.^18^	10x Visium
Mouse Visual Cortex	Hu et al.^14^	STARmap

**Software and algorithms**		

Scanpy	Wolf et al.^28^	https://github.com/scverse/scanpy
STAGATE	Dong and Zhang^19^	https://github.com/QIFEIDKN/STAGATE_pyG
CCST	Li et al.^16^	https://github.com/xiaoyeye/CCST
SEDR	Fu et al.^18^	https://github.com/JinmiaoChenLab/SEDR
SpaGCN	Hu et al.^14^	https://github.com/jianhuupenn/SpaGCN
BayesSpace	Zhaoet al.^17^	https://github.com/edward130603/BayesSpace
ADEPT	This paper/GitHub	https://github.com/maiziezhoulab/ADEPT

### Resource availability

#### Lead contact

Further information and requests for resources and code should be directed to and will be fulfilled by the lead contact, Prof. Xin Maizie Zhou (maizie.zhou@vanderbilt.edu).

#### Materials availability

This study did not generate new unique reagents.

### Method details

#### Data preprocessing

We are using multiple datasets for this study, including 8 samples from the human dorsolateral prefrontal cortex (DLPFC) 10x Visium dataset,^26^ the 10x Visium spatial transcriptomics data of human breast cancer, and the mouse visual cortex STARmap data.^6^ When using these datasets, spots that are outside the main tissue area and spots containing barcodes without annotation are first removed. These outlier spots have extremely low sequencing quality and do not have any adjacent neighbors. Then, raw gene expression data are log-transformed and normalized according to library size using the SCANPY package.^28^ Next, to control and improve the overall expression data quality, ADEPT uses an empirically determined, non-zero rate of 0.14 as a threshold to screen out genes when the total count across cells of the gene does not meet a specific minimum number. This specific minimum number varies for different types of ST datasets or different batches. We chose this empirical threshold because high-quality ST datasets such as the breast cancer dataset have an original non-zero rate close to this value. Since this is an initial preprocessing step, we also did not wish to set this threshold too high to remove too many genes. ADEPT then applies the minimum gene count filtering to further filter low-quality genes. The graph structure is built with the k-nearest-neighbor (kNN) algorithm, which is typically exploited to construct a graph when there are no explicit edge relationships provided. In ADEPT, k is set to 6 as default. This hyperparameter is not very sensitive across different datasets. In the end, the inputs for the model are the list of edges and the node features, which are major components for any graph-based neural network.^31^

#### Graph attention autoencoder

We design the graph attention autoencoder consisting of three core parts: the encoder, the decoder, and the graph attention layer.^32^ In order to strengthen the connection between nodes that are represented by similar expression profiles and to smooth out the clustering result, ADEPT applies the attention layer mechanism, which is widely used to compute the weight between node pairs. These weights indicate the different contributions of each neighbor used in the aggregation process. Based on node embedding, the weights of edges are automatically calculated. Therefore, this model relies less on the initial edge weight and enables learning of the relationships between nodes by implicitly taking consideration of spot similarity, rather than explicitly calculating the weight with heuristic methods (e.g., in SpaGCN^14^).

The encoder’s input consists of the normalized gene expression profile and the edge lists. The encoder then generates node embeddings by aggregating information from all neighbors. We denote $h_{u}^{\left(0\right)}$ as the normalized expression feature of spot *u*. By treating expression profiles as the initial spot embedding, the $l^{\text{th}}$ encoder layer generates the embedding of spot *u* in layer *l* as follows:

(Equation 1) $$h_{u}^{\left(l\right)}=\epsilon \left(\sum_{v\in N_{u}}\alpha_{uv}^{\left(l\right)}\left(W^{\left(l\right)}h_{v}^{\left(l-1\right)}\right)\right)$$

where $\alpha_{uv}^{\left(l\right)}$ is the attention coefficient, *ε* is is an exponential linear unit (ELU),^33^$N_{u}$ denotes all the neighbors of node *u* including *u* itself, $h_{v}^{\left(l-1\right)}$ denotes the embedding of node *v* in the l−1 layer, $a^{\left(l\right)}$ is another learnable parameter, and $W^{\left(l\right)}$ is the matrix of trainable parameters in $l^{\text{th}}$ layer. The attention coefficient $\alpha_{uv}^{\left(l\right)}$ of layer *l* for every node pair (u,v) in the encoder is used to measure the importance of the neighboring node *v* towards learning a higher quality representation of node *u* on graph*G*. This is calculated by Equations 2 and 3 where ⊕ is the concatenation operation and *σ* is a sigmoid activation function.

(Equation 2) $$g_{uv}^{l}=W^{\left(l\right)}h_{u}^{\left(l-1\right)}⊕W^{\left(l\right)}h_{v}^{\left(l-1\right)}$$

(Equation 3) $$\alpha_{uv}^{\left(l\right)}=\frac{\exp\left(\sigma ,\left(a^{{\left(l\right)}^{T}},g_{uv}^{\left(l\right)}\right)\right))}{\sum_{v^{\prime }\in N_{u}}\;\exp\left(\sigma \left(a^{{\left(l\right)}^{T}}g_{uv^{\prime }}^{\left(l\right)}\right)\right)}$$

The output of the *L*-layer encoder $h_{u}^{\left(L\right)}$ is the final embedding of spot *u* and represents the hidden embedding with the lowest dimension. The decoder attempts to reconstruct the normalized expression profile for each spot *u* (i.e., $h_{u}^{\left(0\right)}$) given the latent embeddings of the encoder. More specifically, the output of the encoder is given as the input to a *L*-layer decoder (i.e., ${\hat{h}}_{u}^{\left(0\right)}=h_{u}^{\left(L\right)}$), with the $l^{\text{th}}$ layer of the decoder (from the perspective of spot *u*) defined as:

(Equation 4) $${\hat{h}}_{u}^{\left(l\right)}=\varepsilon \left(\sum_{v\in N_{u}}{\hat{\alpha }}_{uv}^{\left(l\right)}\left({\hat{W}}^{\left(l\right)}{\hat{h}}_{v}^{\left(l-1\right)}\right)\right)$$

where ${\hat{\alpha }}_{uv}^{\left(l\right)}$ is the decoder attention coefficient which is calculated similarly as $\alpha_{uv}^{\left(k\right)}$ in the encoder, and ${\hat{W}}^{\left(l\right)}$ is the matrix of trainable parameters in $l^{\text{th}}$ layer. Ultimately, the output of the decoder, ${\hat{h}}_{u}^{\left(L\right)}$, is trained via updating the parameters of the encoder and decoder layers in an attempt to reconstruct the normalized expression profiles (i.e., $h_{u}^{\left(0\right)}$).

#### Differentially expressed gene lists selection by non-zero rate

As mentioned in the section describing the overall pipeline of ADEPT, several differentially expressed gene (DEG) lists are selected from the initial clusters relying on a range of non-zero rates from 0.3 to 0.4. We chose this empirical threshold range to maintain an optimal number of DEGs. Within this range, ADEPT generates a specific number of DEGs and selects multiple DEG lists that meet the non-zero rate range threshold. Across this range of non-zero rates, ADEPT selects a small proportion of differentially expressed genes and determines if the data quality for each selection is acceptable. Across different types of datasets, we maintain the same range of non-zero rates, resulting in different DEG list selections for the next imputation step. ADEPT performs a Mann-Whitney U test^34^ on each cluster to determine genes with an expression that differs significantly between clusters. To analyze each cluster, the gene expression data are sorted into two groups, with one group containing the values from the cluster to be analyzed and the other group containing the values from all other clusters. Mann-Whitney U tests are implemented by ranking the expression values associated with each spot, one gene at a time.^35^

The ranks for each group are summed and these sums are used to determine the likelihood that gene expression among groups is different. This is performed using Equations 5 and 6, where $n_{x}$ represents the number of spots in the cluster to be analyzed, $n_{y}$ represents the number of spots in all other clusters, $R_{x}$ represents the sum of ranks for the cluster to be analyzed and $R_{y}$ represents the sum of ranks for all other clusters.

(Equation 5) $$U_{x}=\frac{n_{x}\left(n_{x}+1\right)}{2}-R_{x}$$

(Equation 6) $$U_{y}=\frac{n_{y}\left(n_{y}+1\right)}{2}-R_{y}$$

The minimum value between $U_{x}$ and $U_{y}$ is used to find the p-value of each gene via Mann-Whitney tables.^35^ Genes with the lowest p-values are the most statistically significant between groups. When using this method, there is a trade-off for the number of DEGs selected. Since all the DEGs are selected sequentially from the top ranking to the bottom, more features will be kept when we choose to select more DEGs. However, keeping more DEGs introduces increasingly more noise and a higher dropout rate. On the other hand, if we choose to keep the smallest amount of DEGs possible, many useful features will be sacrificed. Here, we thus introduce a DEG candidates selection step, which aims to optimize the number of DEGs which are kept for each cluster by optimizing the range of the non-zero rates after performing DEG selection. Once several different DEG lists are selected based on the initial clustering results, they will be utilized for imputation and final clustering.

#### Expression matrix imputation

At this stage, ADEPT has selected several DEG lists based on non-zero rates and extracted multiple DEG-based matrices. We then impute these matrices and merge them for robust estimation, to minimize the variance of the final clustering result. The strategy of our imputation method is to use the average expression value of the gene within the same cluster and the pseudo-labels that are obtained from our initial clustering result to complete the dropout entries.

Specifically, we denote $X\in R^{g\times c}$ as a gene expression matrix, where *g* is the number of genes and *c* is the number of spots. The ${\left(i,j\right)}^{\text{th}}$ component of X is represented as $x_{ij}$. The estimated value of a dropout event is given by averaging the gene expression values across all spots for each non-zero gene within the same cell cluster:

(Equation 7) $$E\left(x_{ij}\right)=\frac{\sum_{j^{\prime }\in cluster_{n}}x_{ij^{\prime }}}{\left\|cluster_{n}\right\|}$$

$x_{ij}$ denotes $i^{\text{th}}$ gene dropout of spot *j*. $j^{\prime }\in cluster_{n}$ refers to all other spots within the same cluster that have values in their $i^{\text{th}}$ gene, while$\left\|cluster_{n}\right\|$ indicates the size of ${cluster}_{n}$ excluding those spots with dropout in $i^{\text{th}}$ gene.

Additionally, we denote $C_{1}$, $C_{2}$, ⋯, $C_{K}$ as different clustering results based on different selected DEG lists. The $E\left(x_{ij}|C_{K}\right)$ was computed for each clustering result $C_{1}$, $C_{2}$, ⋯, $C_{K}$. Finally, ADEPT estimates the final imputation for dropout events $x_{ij}$, and $E\left(x_{ij}\right)$, by computing the average of the estimated results from all different DEG lists:

(Equation 8) $$E\left(x_{ij}\right)=\frac{1}{K}\sum_{k=1}^{K}E\left(x_{ij}|C_{k}\right)$$

The imputed expression matrix contains all genes that occur in either of the *K* DEG lists at least once.

### Quantification and statistical analysis

Statistical details and software used for various types of data analyses in this work are cited in the appropriate sections in the STAR Methods. The agreements between true cluster labels and clustering results from spatial transcriptomics (ST) data without or with imputation were calculated using Adjusted Rand Index (ARI), Fawlkes Mallows Score (FMS), and purity score. The layer marker genes expression and spatial trajectory analyses were performed by using the Scanpy package.

## Data Availability

• All data reported in this paper will be shared by the lead contact upon request. • All original code has been deposited on our GitHub repository (https://github.com/maiziezhoulab/ADEPT) and is publicly available. • Any additional information required to reanalyze the data reported in this paper is available from the lead contact upon request.
