# Supplementary material for: ADEPT: Autoencoder with differentially expressed genes and imputation for robust spatial transcriptomics clustering
Source: iScience. 2023 May 3;26(6):106792. doi: 10.1016/j.isci.2023.106792 (PMC10205785; doi:10.1016/j.isci.2023.106792)
Supplement: Document S1. Tables S1–S3 [file mmc1.pdf]

**iScience, Volume *26***

## Supplemental information

# ADEPT: Autoencoder with differentially expressed genes and imputation for robust spatial transcriptomics clustering

**Yunfei Hu, Yuying Zhao, Curtis T. Schunk, Yingxiang Ma, Tyler Derr, and Xin Maizie Zhou**

# ADEPT: autoencoder with differentially expressed genes and imputation for a robust spatial transcriptomics clustering

## Supplementary Information

Supplementary Table 1: **The statistic table of DEG lists and their corresponding non-zero rate for each dataset or section. Related to STAR Methods section "Control Data Quality by Non-zero Rate"** For DLPFC sections, ADEPT utilized top 50, 100, ..., and 500 as the DEG candidate lists. In each entry, these two values refer to the total number of DEGs for all clusters and the corresponding non-zero rate for the DEG-based expression matrix. The bold entries represent the corresponding non-zero rates that are within the threshold of 0.3 - 0.4, which are used for the next imputation step of ADEPT. For the breast cancer (BC) dataset, ADEPT utilized top 100, 200, ..., and 600 as the DEG candidate lists to estimate this step. For the STARmap dataset, ADEPT utilized top 25, 50, ..., and 200 as the DEG candidate lists to estimate this step.

| Top $N$ DEG list (DLPFC) | 151507 | 151508 | 151509 | 151510 | 151673 | 151674 | 151675 | 151676 | Top $N$ DEG list (BC) | BC1 | Top $N$ DEG list (STARmap) | STARmap |
|---|---|---|---|---|---|---|---|---|---|---|---|---|
| $N$=50 | 275/0.45 | 280/0.43 | 280/0.48 | 290/0.42 | 290/0.49 | 280/0.60 | 290/0.44 | 290/0.50 | $N$=100 | 1200/0.58 | $N$=25 | **150/0.36** |
| $N$=100 | 550/0.40 | **550/0.36** | 585/0.40 | **570/0.38** | 590/0.43 | 560/0.53 | **580/0.39** | 590/0.43 | $N$=200 | 2240/0.54 | $N$=50 | **300/0.31** |
| $N$=150 | **840/0.34** | **830/0.32** | **840/0.35** | **855/0.32** | **880/0.39** | 810/0.47 | **860/0.35** | **900/0.38** | $N$=300 | 3300/0.51 | $N$=75 | 430/0.29 |
| $N$=200 | **1075/0.33** | **1100/0.29** | **1105/0.31** | 1120/0.29 | **1120/0.38** | 1120/0.43 | **1120/0.33** | **1190/0.36** | $N$=400 | 4300/0.47 | $N$=100 | 540/0.28 |
| $N$=250 | **1330/0.31** | 1370/0.27 | 1390/0.29 | 1400/0.28 | **1385/0.37** | 1400/0.41 | **1420/0.32** | **1470/0.34** | $N$=500 | 5200/0.46 | $N$=150 | 750/0.25 |
| $N$=300 | 1600/0.29 | 1650/0.25 | 1660/0.27 | 1680/0.26 | **1600/0.36** | **1680/0.39** | 1680/0.29 | 1750/0.29 | $N$=600 | 6150/0.45 | $N$=200 | 870/0.24 |
| $N$=350 | 1890/0.27 | 1930/0.24 | 1930/0.26 | 1925/0.25 | **1770/0.35** | **1840/0.37** | 1980/0.28 | 2030/0.29 | - | - | - | - |
| $N$=400 | 2140/0.26 | 2175/0.23 | 2195/0.25 | 2210/0.24 | **2170/0.32** | **2260/0.33** | 2270/0.27 | 2320/0.28 | - | - | - | - |
| $N$=450 | 2400/0.25 | 2430/0.22 | 2425/0.25 | 2415/0.24 | 2410/0.29 | **2550/0.32** | 2510/0.26 | 2545/0.27 | - | - | - | - |
| $N$=500 | 2630/0.24 | 2665/0.22 | 2660/0.24 | 2675/0.22 | 2695/0.27 | 2810/0.29 | 2795/0.25 | 2820/0.26 | - | - | - | - |

Supplementary Table 2: **Ranking table of ADEPT and other five baseline methods for robustness testing. Related to STAR Methods section "ADEPT Demonstrates Robust Clustering Performance Across Different ST Datasets"** The rankings from 1 to 6 are listed by each tool for each dataset. The sums of rankings and the average of total rankings are calculated in the end for each method.

| Methods | 151507 | 151508 | 151509 | 151510 | 151673 | 151674 | 151675 | 151676 | BC1 | STARmap | Sum of rankings | Average ranking |
|---|---|---|---|---|---|---|---|---|---|---|---|---|
| ADEPT | 2 | 1 | 1 | 1 | 1 | 1 | 1 | 1 | 2 | 1 | 12 | 1.2 |
| STAGATE | 1 | 2 | 2 | 2 | 2 | 2 | 2 | 2 | 6 | 2 | 23 | 2.3 |
| CCST | 4 | 3 | 4 | 3 | 6 | 5 | 3 | 3 | 3 | 6 | 40 | 4.0 |
| SEDR | 5 | 5 | 5 | 4 | 3 | 3 | 4 | 4 | 4 | 5 | 42 | 4.2 |
| SpaGCN | 3 | 4 | 3 | 3 | 4 | 4 | 5 | 5 | 1 | 3 | 35 | 3.5 |
| BayesSpace | 6 | 6 | 6 | 5 | 5 | 6 | 6 | 6 | 5 | 4 | 55 | 5.5 |

Supplementary Table 3: **Average runtime results of 10x Visium and STARmap datasets. Related to STAR Methods section "Experimental Setup and Default Parameters for ADEPT"** For all experiments, we used Intel(R) Xeon(R) W-2195 CPU with 2.30GHz frequency, 1 Quadro RTX 8000 GPU with 48GB memory, and Ubuntu 20.04.3 LTS (Focal Fossa) operation system. The CPU has 18 cores with 36 threads.

| Methods | 10x Visium | STARmap |
|---|---|---|
| ADEPT | 17 mins | 1.5 mins |
| STAGATE | 2 mins | <1 min |
| CCST | 38 mins | 10 mins |
| SEDR | 5 mins | 1.5 mins |
| SpaGCN | 3 mins | 1.5 mins |
| BayesSpace | 3 mins | 2 mins |
